# Let's do it!

**DOI:** 10.3402/mehd.v23i0.20248

**Published:** 2012-12-27

**Authors:** Tore Midtvedt

The shift of *Microbial Ecology in Health and Disease* to an Open Access publication model and a dedicated publishing house has now started to bear fruit.

The so-called Impact Factor is one of several ranking indicators that are important for the development of a scientific journal, and I am therefore pleased to inform our authors and readers that, in July 2012, *Microbial Ecology in Health and Disease* was entered into online evaluation by Thomson Reuters (ISI), the company offering this citation indexing service. However, it will probably take 3–4 years before the journal actually obtains an official Impact Factor due to the way in which this factor is calculated. But now the process has started. Personally, my first priority is to publish high-quality research papers that will be *read* by researchers all over the world; if these papers are also *cited* by these researchers, I will only be happy.

For a long time we have wanted to have the journal included in PubMed Central/PubMed and the journal is now finally in for evaluation by the Selection Group at the National Library of Medicine (NLM), and we hope for a verdict soon. PubMed Central (PMC) is the free archive of biomedical and life sciences journal literature at the U.S. National Institutes of Health's NLM, and is designed to provide permanent access to all of its content, even as technology evolves and current digital literature formats potentially become obsolete. Should *Microbial Ecology in Health and Disease* be accepted for inclusion, PMC will submit a citation to PubMed as soon as an article in the journal is live in PMC, i.e. a couple of days after it has been published on the journal's website.

The key problem of this long waiting has not been because of the quality of the content we publish but because the inflow of manuscripts is too slow. In fact, as Chief Editor, that is at present my major concern. In particular, I am somewhat surprised that so few SOMED members contribute with their work. After all it is *their* journal, and they are only charged a publication fee of €300 as compared to the non-member fee of €800. In fact, €300 is a comparatively small fee for publication in an Open Access journal. To get *Microbial Ecology in Health and Disease* to now lift I therefore encourage you to submit at least one paper to the journal in the near future – either from your own group or for the wider network you are working within – so that the evaluations currently going on at NLM and Thomson Reuters, as described above, may eventually turn out to the journal's advantage.

Then I want to share some new ideas with you:

There is still a lot of turbulence around the word “probiotics”. Regulatory rules vary considerably worldwide, thus creating problems for researchers as well as producers. In the months to come – and before the next SOMED conference in Kosice in September 2013 – letters from you describing your experiences and/or opinion of this subject are highly appreciated.

I also want to receive proposals for new thematic clusters. The cluster of articles around the topic “Autism Spectrum Disorders” turned out to be a great success and has encouraged me to embark on new special issues – it could be either proceedings from a conference as was the case with the Autism collection of papers, or simply by inviting researchers in a particular field to contribute a paper on a clearly defined theme. “Probiotics” and/or “Fecal transplantation” might be two suitable topics. Ideas for other special issues are very welcome.

I also want to encourage you to re-view any old documents with “unpublished results” that you have jotted down over the years. I am sure many of you will find some interesting notes revolving around problems that still are not solved. Surely, your experiments may have been done by “yesterday's technology” and may not deserve to be published *in toto* any longer; but your old work might turn out to be of interest when applying today's technology to these as yet unsolved problems. Therefore, I kindly ask you to submit letters or notes to be published under the heading “Notes from the past for the future”. I will try to put them into a proper historical perspective if and when they are published.

To conclude, I want to thank you for your continued interest in *Microbial Ecology in Health and Disease*. I hope you will submit one or more papers soon, and perhaps even respond to one or more of the above suggestions to make the journal an even more interesting and lively forum for research and debate in our field of expertise. Only together can we make 2013 the best year ever for *Microbial Ecology in Health and Disease*.

Karolinska Institute, November 2012 *Tore Midtvedt* Editor-in-Chief

